# Understanding high and low patient experience scores in primary care: analysis of patients’ survey data for general practices and individual doctors

**DOI:** 10.1136/bmj.g6034

**Published:** 2014-11-11

**Authors:** Martin J Roberts, John L Campbell, Gary A Abel, Antoinette F Davey, Natasha L Elmore, Inocencio Maramba, Mary Carter, Marc N Elliott, Martin O Roland, Jenni A Burt

**Affiliations:** 1 University of Exeter Medical School, St Lukes Campus, Exeter EX1 2LU, UK; 2 Cambridge Centre for Health Services Research, Institute of Public Health, Forvie Site, University of Cambridge School of Clinical Medicine, Box 113 Cambridge Biomedical Campus, Cambridge CB2 0SR, UK; 3 RAND Corporation, 1776 Main Street, PO Box 2138, Santa Monica, CA 90407-2138, USA

## Abstract

**Objectives** To determine the extent to which practice level scores mask variation in individual performance between doctors within a practice.

**Design** Analysis of postal survey of patients’ experience of face-to-face consultations with individual general practitioners in a stratified quota sample of primary care practices.

**Setting** Twenty five English general practices, selected to include a range of practice scores on doctor-patient communication items in the English national GP Patient Survey.

**Participants** 7721 of 15 172 patients (response rate 50.9%) who consulted with 105 general practitioners in 25 practices between October 2011 and June 2013.

**Main outcome measure** Score on doctor-patient communication items from post-consultation surveys of patients for each participating general practitioner. The amount of variance in each of six outcomes that was attributable to the practices, to the doctors, and to the patients and other residual sources of variation was calculated using hierarchical linear models.

**Results** After control for differences in patients’ age, sex, ethnicity, and health status, the proportion of variance in communication scores that was due to differences between doctors (6.4%) was considerably more than that due to practices (1.8%). The findings also suggest that higher performing practices usually contain only higher performing doctors. However, lower performing practices may contain doctors with a wide range of communication scores.

**Conclusions** Aggregating patients’ ratings of doctors’ communication skills at practice level can mask considerable variation in the performance of individual doctors, particularly in lower performing practices. Practice level surveys may be better used to “screen” for concerns about performance that require an individual level survey. Higher scoring practices are unlikely to include lower scoring doctors. However, lower scoring practices require further investigation at the level of the individual doctor to distinguish higher and lower scoring general practitioners.

## Introduction

Public reporting of performance measures is increasingly the norm in healthcare systems.^1^ Forming part of the drive for continuous quality improvement, the disclosure of results of assessments at either provider or individual level is believed to increase accountability and public engagement.^2^ A recent US report highlighted the important contribution that listening to, and acting on, patients’ feedback can potentially make to efforts to improve healthcare.^3^ New developments in the English National Health Service highlight the embedding of public assessment of performance within the regulation of the healthcare system, including NHS England’s consultation on the production of general practice league tables and the Care Quality Commission’s parallel development of a rating system for primary care.^4^ ^5^ An increasingly transparent healthcare system, in which providers are publicly gauged against performance targets, is regarded by policy makers as essential to enabling patients to make informed choices about the care they receive.^6^ Consequently, patients’ feedback on healthcare services is now gathered in the United States, Canada, Europe, Australia, China, and elsewhere.

This increased emphasis on patients’ feedback in healthcare is reflected in extensive investment in both collection and use of patients’ experience data to evaluate providers’ performance. In the United Kingdom, for example, the NHS Outcomes Framework 2013/14 requires that “people have a positive experience of care.” For primary care, this is assessed on the basis of responses to the English national GP Patient Survey of patients’ experiences with their general practitioner surgery.^7^ This major source of patient experience data, currently administered to over 2 million people annually, is also the source for general practices’ performance scores compiled for and advertised on websites such as NHS Choices and Compare.^8^ ^9^ ^10^ Similar internet based formats for reporting patient experience data, whether generated by governments, patient groups, or commercial organisations, are emerging across the globe.^11^ ^12^ ^13^

Several causal pathways for achieving improvements in providers’ performance through the release of publicly reported performance data have been proposed.^1^ ^2^ ^14^ Some invoke market-like selection, claiming that patients will modify their choice of provider by using publicly available data, such as that provided by patient experience websites.^11^ ^12^ ^13^ ^14^ Evidence to support this pathway is, however, weak.^2^ A more likely mechanism driving improvement in performance in response to the publication of performance data is health professionals’ concern for reputation, in which peer comparison motivates individuals and organisations to improve their care.^1^ ^2^

Irrespective of its potential to stimulate change, the publication of performance data is central to the openness and transparency that are seen as essential to a safe, equitable, patient centred healthcare system.^15^ Thus, regardless of any effect on quality improvement, such initiatives are likely to be here to stay.^2^ In refining the information made public, performance data need to be accurate and relevant to all potential users. The US based Robert Wood Johnson Foundation has noted that although patients “prefer to see comparative information for individual providers rather than practices or groups,” this is often not done in practice.^16^ Currently, however, the focus is moving from the publication of performance data at an organisational level to that of individual doctors. In the United Kingdom, for example, patients referred to the cardiology service at the South Manchester Hospitals Trust may go online to view both mortality and patient experience data for each cardiologist or cardiac surgeon.^17^ However, in English primary care, nationally collected performance data remains at the level of the practice, not the practitioner. The practice level aggregation of data from the GP Patient Survey, used to derive practices’ performance indicators, potentially masks considerable variation in performance among individual general practitioners, thereby inappropriately advantaging or disadvantaging particular doctors. Current indicators may consequently fail to provide users, providers, or commissioners with an accurate assessment of performance within a practice.

We aimed to explore the extent to which aggregated practice scores may mask variation in individual performance. We focused on patients’ assessments of doctors’ communication skills: interpersonal aspects of care are a key driver of overall patients’ satisfaction and are a major component of the GP Patient Survey used to derive the “overall patient experience of care” scores advertised for each general practice on NHS Choices (alongside waiting to be seen, opening hours, and consultations with a nurse).^9^ ^18^

## Methods

### Sample and data collection

We invited a stratified random sample of general practices in six areas of England (Cornwall, Devon, Bristol, Bedfordshire, Cambridgeshire, and North London) to participate in the study. Our aim was to recruit a set of practices that varied substantially in ratings for doctors’ consultation skills. Practices were classified at baseline according to their case mix adjusted score on a composite of seven items relating to doctor-patient communication in the 2009/10 English GP Patient Survey. We used linear regression models adjusting for patients’ age, sex, ethnicity, deprivation score, and self rated health to adjust for case mix.^19^ We aimed to recruit 15 practices with scores in the lowest 25% of all practices, five practices scoring in the middle quarter (37.5th to 62.5th centile), and five practices scoring in the highest quarter (above the 75th centile). Eligible practices had at least two registered general practitioners working at least four sessions a week (0.4 full time equivalent), excluding trainees and short term locums. We drew a stratified random sample, stratifying by the communication score banding, general practitioner head count, deprivation index, and geographical location. We approached eligible practices in a randomised order until the quota for each stratum was obtained.

Data collection took place between October 2011 and June 2013. We did a postal survey of patients who had recently attended a face-to-face consultation with a participating general practitioner. For each wave of the survey, we extracted from electronic records a list of face-to-face doctor-patient consultations held during the previous three weeks. Practices screened each list for recent deaths, recent bereavement, terminal illness, and mental incapacity: all such patients were excluded. Practices sent the remaining patients a patient experience survey based on the national GP Patient Survey, asking them about access, waiting times, opening hours, and continuity and interpersonal aspects of care. The questionnaire also included questions about sociodemographic information including age, sex, ethnicity, and self rated health. In completing the seven interpersonal care items and one confidence and trust item, patients were asked to think back to a consultation with a specified doctor on a specified date (corresponding to the consultation identified from the extracted records). Patients who attended multiple consultations were sent only one questionnaire, relating to their most recent consultation at the point of data extraction. One reminder was sent to patients who did not respond within three weeks; we accepted returned questionnaires up to 100 days after the initial mail out.^20^ We repeated the survey cycle in each practice until either we had received 50 or more completed questionnaires for each participating general practitioner or three cycles had been completed. Fifty questionnaires are sufficient to obtain reliable mean communication scores for comparable patient feedback instruments.^20^ ^21^ Return of a completed questionnaire was taken to indicate patients’ consent to participate in the study.

### Outcome measures

Our primary outcome measure was a communication score for the doctor from each respondent. We derived this as the mean rating across the seven communication items (questions 22a to 22g; see supplementary material) among patients providing four or more informative responses. The first of our five secondary outcomes asked patients about their confidence and trust in the doctor they saw. We also analysed four secondary measures relating to practice level variables: patients’ ratings of overall satisfaction with care at the practice, helpfulness of the receptionists, cleanliness of the facilities, and ease of entry to the building. All items were rescaled linearly from 0 (least favourable) to 100 (most favourable).

### Statistical analysis

We described the sex balance, proportion of doctors who trained in the United Kingdom, and mean time since registration in the general practitioner sample, together with questionnaire response rates and the intervals between consultations with patients, mail out of questionnaires, and return of questionnaires. We tested whether consultation to mail out intervals were associated with response to questionnaires by using a two sample *t* test.

In our study design, groups of individual patients’ scores are associated with (nested within) individual general practitioners, and groups of general practitioners are associated with individual practices. Although some variance in patients’ scores can be attributed to individual experiences (including personal expectation, outlook, and the variable performance of general practitioners between patients), some of the variance in patients’ scores is likely to be attributable to general practitioners, with some doctors performing better, on average, than others. Furthermore, aspects of the practices beyond the general practitioners (for example, reception staff, opening hours) may account for some variation. Our aim was to assess the extent to which aggregated practice scores may mask within practice variation in performance by individual general practitioners. We used three-level mixed-effects hierarchical linear models to estimate the amount of variance in each of the outcome measures that could be attributed to differences between the practices, to differences between the doctors within each practice, and to the patients and other residual sources.^22^ Such models represent an extension of analysis of variance based approaches, taking account of the inherent hierarchical structure of the data: patients are clustered within doctors, who in turn are clustered within practices.

We adjusted all models for four self reported patients’ attributes previously shown to be important predictors of reported patient’s experience: the patient’s sex, age (eight ordinal categories), ethnicity (16 categories), and self reported health status (five ordinal categories).^23^ We expressed the practice, doctor, and patient related variance components from each model as percentages of the total variance and used the “best linear unbiased predictors” of the practice and doctor effects to provide estimates of the mean score for each doctor on each of the outcome measures.^24^ Corresponding estimates of the mean scores for each practice came from additional models, which omitted random effects for doctors. We described the variation in the general practitioners’ and practices’ mean scores and used simple correlation analysis to investigate the association between the practices’ mean score and the within practice standard deviation of the general practitioners’ mean scores. We used the variance components from each model to estimate the number of patients’ scores per doctor needed to achieve a reliability of at least 0.7 or 0.8 for the doctor’s mean score (see appendix for the formula used). Whereas a reliability of 0.8 or higher is desirable for moderate to high stakes assessments,^25^ a threshold of 0.7 is regarded as acceptable in patients’ assessments of doctors’ performance in some contexts.^26^ We used Stata SE version 10.1 for data analysis.

## Results

Of 59 practices initially approached, six were found to be ineligible, nine declined participation, and 19 had not responded by the time we achieved our quota of 25 participating practices. Table 1 provides brief profiles of the participating practices. There were 105 participating doctors (mean 4.2 (range 2-8) doctors per practice), of whom 46% were female and 80% were trained in the United Kingdom. Average time since registration with the General Medical Council was 19.5 (range 4-38) years. Table 2 shows respondents’ demographics. The mean interval between the patient’s consultation date and the mail out of their questionnaire was 16.6 (SD 6.0) days. We found no evidence that the length of this interval was related to the likelihood of the patient returning a completed questionnaire (two sample *t* test, P=0.157). The overall questionnaire response rate was 50.9% (7721/15 172), ranging from 23.6% to 80.7% for individual general practitioners and 24.1% to 75.5% for practices (table 1). The target of 50 returned questionnaires was achieved for 92 (87.6%) of the general practitioners. The mean interval between the patient’s consultation date and our receipt of their completed questionnaire was 35.3 (SD 15.5) days.

**Table 1 tbl1:** Practice profiles and questionnaire response rates

Setting	Banding on 2009/10 GPPS communication score*	GP head count	Participating doctors	List size (000s)	Deprivation index†	Overall response rate (%)
Inner city	Low	2	2	6.9	26.6	37.9
Inner city	Low	3	3	5.1	48.5	36.8
Inner city	Low	4	4	5.1	36.6	37.8
Inner city	Low	5	4	7.8	26.1	50.5
Inner city	Low	8	6	8.7	32.4	43.5
Inner city	Middle	2	2	2.5	30.1	47.0
Inner city	Middle	3	3	5.4	13.7	67.7
Inner city	Middle	6	6	8.0	39.4	32.0
Urban	Low	2	2	3.5	15.2	71.0
Urban	Low	2	2	2.9	22.2	58.9
Urban	Low	2	2	3.2	29.6	24.1
Urban	Low	3	3	6.6	15.1	55.8
Urban	Low	4	4	4.1	18.3	59.3
Urban	Low	5	5	12.0	27.6	58.9
Urban	Low	5	5	6.0	19.3	52.6
Urban	Low	7	6	9.7	20.0	53.8
Urban	Low	8	7	16.5	14.4	45.1
Urban	Low	9	8	11.8	16.4	48.1
Urban	Middle	3	3	5.3	20.8	67.8
Urban	High	6	5	8.5	22.1	47.2
Urban	High	8	8	14.2	18.9	64.4
Rural	Middle	5	4	5.1	23.1	60.5
Rural	High	3	2	2.4	18.9	49.8
Rural	High	4	4	5.4	11.5	75.5
Rural	High	5	5	9.1	4.8	71.7
All	—	114	105	—	—	50.9

GPPS=General Practice Patient Survey.

*Low=below 25th centile; middle=between 37.5th and 62.5th centiles; high=above 75th centile.

†Average taken across practice population; these scores underlie figures reported by Public Health England at http://fingertips.phe.org.uk/profile/general-practice.

**Table 2 tbl2:** Demographic profile of responding patients (n=7721)

	No (% of non-missing)
**Sex**	
Female	4785 (62.4)
Male	2882 (37.6)
Missing	54
**Age (years)**	
<18	5 (0.1)
18-24	249 (3.2)
25-34	786 (10.3)
35-44	983 (12.8)
45-54	1150 (15)
55-64	1474 (19.2)
65-74	1550 (20.2)
75-84	1171 (15.3)
≥85	299 (3.9)
Missing	54
**Ethnicity**	
White British	6138 (81.5)
White Irish	132 (1.8)
Any other white background	459 (6.1)
Mixed white and black Caribbean	23 (0.3)
Mixed white and black African	10 (0.1)
Mixed white and Asian	18 (0.2)
Any other mixed background	19 (0.3)
Asian or Asian British—Indian	169 (2.2)
Asian or Asian British—Pakistani	55 (0.7)
Asian or Asian British—Bangladeshi	71 (0.9)
Any other Asian background	72 (1)
Black or black British—Caribbean	95 (1.3)
Black or black British—African	161 (2.1)
Any other black background	9 (0.1)
Chinese	45 (0.6)
Any other ethnic group	57 (0.8)
Missing	188
**Health**	
Poor	714 (9.5)
Fair	1827 (24.3)
Good	2502 (33.2)
Very good	1961 (26.1)
Excellent	523 (6.9)
Missing	194

By excluding questionnaires with fewer than four informative responses to the seven communication items, we calculated communication scores for 7429 (96.2%) responding patients. The mean communication score was 87.5 (SD 17.8) on a 0-100 scale.

### Main findings

Table 3 shows the variance components for the six outcome measures estimated with the hierarchical models. In all cases, most of the variance in patient level scores was due to differences in ratings of the same doctor by different patients. For both of the doctor specific measures that we investigated (doctors’ communication and trust and confidence in the doctor), the variance due to differences between doctors was greater than that attributable to differences between practices, whereas the reverse was true for the other four, non-doctor specific, measures. For each outcome measure, table 4 shows the number of patients’ ratings needed to achieve the 0.7 and 0.8 reliability thresholds, judged by authorities to represent minimum acceptable thresholds in postgraduate assessment settings.^27^ A substantial majority of doctors received sufficient scores to achieve reliable estimates of performance in communication—all but two of the 105 general practitioners in our sample received at least 27 patients’ communication scores, and all but 10 received 46 or more (overall mean 71 scores per doctor).

**Table 3 tbl3:** Percentages of variance in adjusted mean outcome scores that are attributable to practices, doctors, and patients

Outcome measure	Source of variance		
	Practice	Doctor	Patients and residual error
Communication score	1.8	6.4	91.9
Confidence and trust	0.8	5.2	94.0
Overall satisfaction with surgery	6.0	1.1	92.9
Helpfulness of receptionists	7.3	0.5	92.2
Cleanliness of health centre	10.6	0.3	89.1
Ease of getting into building	1.9	0.4	97.6

**Table 4 tbl4:** Number of patients’ ratings needed to achieve reliability of 0.7 or 0.8 for doctor’s raw and adjusted mean scores

	Communication score	Confidence and trust	Overall satisfaction with surgery	Helpfulness of receptionists	Cleanliness of health centre	Ease of getting into building
**Reliability of raw mean score**						
0.7	21	30	23	25	15	78
0.8	36	51	38	42	26	133
Reliability of adjusted mean score*						
0.7	27	37	31	28	20	97
0.8	46	63	53	48	33	167

*Adjusted for patient’s sex, age, ethnicity, and self reported health status.

Figure 1 shows the estimated mean communication scores for individual doctors and for practices as a whole. It illustrates the extent to which the variation in mean communication scores between individual doctors (within practices) was greater than the variation between practices and suggests that within practice variability in doctors’ scores was greater in the lower scoring practices. Further analysis confirmed this: the within practice standard deviation of general practitioners’ mean communication scores was negatively correlated with the practice’s mean communication score (Pearson’s *r*=−0.505; P=0.010). Figure 2 shows the adjusted doctor level and practice level mean scores for “cleanliness of the practice buildings” and highlights, in contrast to figure 1, the minimal within practice variability between general practitioners for this non-doctor-specific measure.

**Figure fig1:**
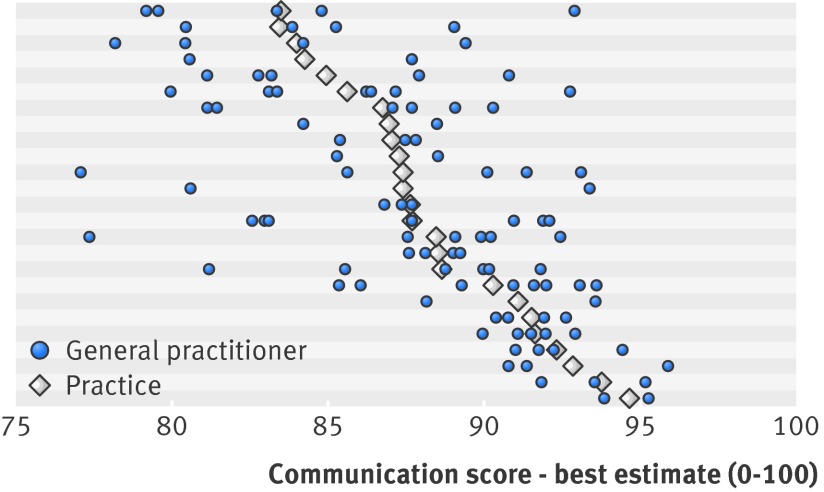
**Fig 1** Mean communication score (best estimate) by practice and doctor. Practices (n=25) are sorted by their mean communication score. Horizontal shading serves only as visual separation of results for different practices. Reliability calculations using variance components showed that achieving acceptable reliability (>0.7) for general practitioners’ adjusted mean communication scores with 27 patients’ scores and good reliability (>0.8) with 46 patients’ scores per doctor is feasible (see appendix). All but 10 of the 105 participating doctors had more than 46 scores; two received less than 27 scores (mean 71 scores per doctor). Data for these doctors was retained in the subsequent modelling, as use of best linear unbiased predictors to estimate doctors’ mean scores has a “conservative” effect. Where sample sizes are smaller, estimated mean scores are drawn closer to practice mean

**Figure fig2:**
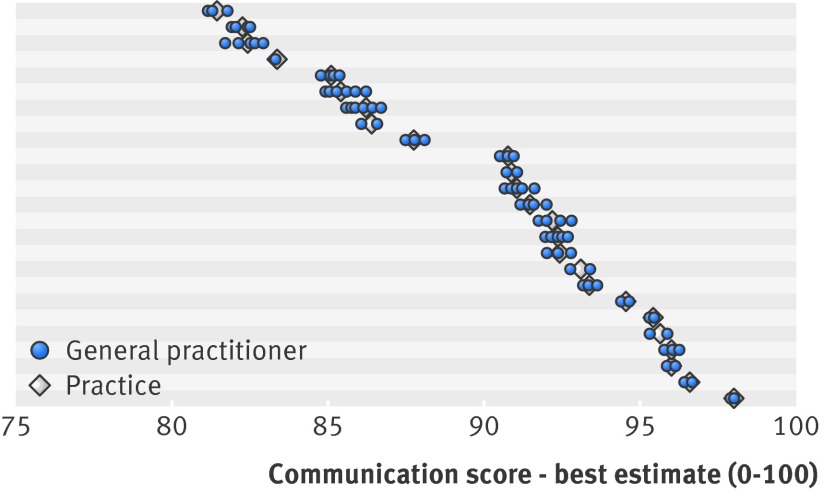
**Fig 2** Mean score for cleanliness of practice building (best estimate) by practice and doctor. Practices (n=25) are sorted by their mean score for cleanliness. Horizontal shading serves only as visual separation of results for different practices

## Discussion

Our results show that measurement of patients’ experience at the practice level can mask considerable variation between doctors within a practice. Our findings suggest that higher performing practices usually comprise higher performing doctors. However, lower performing practices may contain doctors with communication scores ranging from poor to very good. When the focus of patients’ ratings is on non-doctor specific practice related attributes (such as the cleanliness of the facilities), these are measured well at the practice level.

### Strengths and limitations of study

This was a large study, with survey responses from 7721 patients relating to 105 doctors in 25 practices. Our stratified sampling strategy secured participation from doctors delivering care in practices with a range of summary scores for interpersonal skills after adjustment for case mix, and this improves generalisability to wider primary care contexts. Our use of a postal survey resulted in an average delay of just over two weeks between the patient’s consultation and their receipt of the questionnaire—substantially less than the six month reference timeframe adopted in the English GP Patient Survey. Furthermore, we believe a two week delay is unlikely to be a significant source of recall bias and, in any event, would reasonably be expected to affect all doctors equally in this study.

Because of the quota sampling strategy, a simple participation rate for practices could not be calculated. Low response rates are commonly encountered in patient feedback surveys. However, our patient response rate of 51% was considerably higher than the 35% achieved in the most recent published results for the GP Patient Survey (July 2012 to March 2013) and in similar surveys elsewhere.^8^ ^28^ Surveys such as these are typically used to inform national metrics of healthcare performance.^7^ Substantial variation existed in survey response rates between the general practitioners in our study, even after we accounted for the role of chance. Non-response will tend to inflate doctors’ and practices’ scores, but this inflation will be largest when non-response is highest.^29^ Given that the lowest response rates tend to occur for practices with the lower scores, any non-response bias would tend to attenuate the extent of variance between both doctors and practices rather than inflate it. The estimated magnitudes of such effects are small, and we would not expect them to alter the ratios of variance at the practice and doctor levels or affect our conclusions regarding the comparison of doctor and practice level variances.

Our sampling of practices from the lowest, middle, and highest quarter of GP Patient Survey practice level communication scores may mean that our estimate of the total amount of practice level variance could differ slightly from that of the full population; however, we believe that this does not affect our conclusions regarding the relation between practice level scores and the extent of within practice variation. Finally, we were blinded to patients’ postcodes and hence could not adjust our outcome measures for neighbourhood level deprivation. This limitation is unlikely to have biased our results, as we have previously shown that after sex, age, ethnicity, and health status are controlled for (as we did in this study), deprivation has a very small association with patients’ experience.^23^

### Research in context

Whereas Howie and colleagues described the variation within a sample of Scottish practices in respect of doctors’ communication,^30^ several other studies have used hierarchical models to apportion the sources of variance in patients’ feedback in primary care settings.^20^ ^31^ ^32^ ^33^ ^34^ ^35^ ^36^ Few studies have attempted to distinguish the relative contributions of doctors and organisations. In general, these studies concur with our finding that the proportion of variance due to doctors is greater than that due to practices in the case of doctor specific measures and is less in the case of non-doctor specific measures.^32^ ^35^ ^36^ In contrast to these findings, Rodriguez and colleagues found a greater proportion of variance in all types of measure, including physician-patient communication, to be due to sites, medical groups, and primary care service areas (combined) than due to doctors.^33^ Whether a distinction was made between doctors and organisations, all of these studies showed that most of the variance in patient level scores can be attributed to patients and residual sources. Salisbury and colleagues noted that a high proportion of the variance in communication scores in English general practice is attributable to patients and other factors, rather than to practices or individual doctors.^32^ The authors interpreted this as indicating that “so little variation exists at the level of the doctor that the reliability of using this type of measure to assess an individual doctor’s performance is questionable.” This interpretation ignores the fact that doctors (and practices) are assessed not by using the rating provided by a single patient but by using the average of many patients’ ratings. This considerably reduces the “noise” created by variation at the patient level. We suggest that the focus for survey data should be on unit level reliability: the proportion of variance in reporting unit samples’ means (for example, practices’ means or doctors’ means) attributable to true variation between units.^31^ Our results suggest that despite the high proportion of patient level variance in communication scores, for this survey instrument a reliable (>0.8) adjusted mean score for individual doctors can be obtained with 46 patient scores per general practitioner, so that only a small minority of variance in reported doctor level scores is attributable to patients and residual sources. This is in line with our previously published data examining patients’ feedback for the purposes of revalidation.^20^ With sample sizes smaller than this, a trade off must be made between reliability and the utility of conducting individual rather than group level evaluations.^37^

The trade-off between the assessment and reporting of performance indicators at the level of either the organisation or the individual practitioner may be informed by considering both the nature of the indicator and sources of variance. We have shown that, for indicators that are most likely to be under the control of individual practitioners (such as doctor-patient communication), more variance is explained by doctors than by practices. This can be taken as a validation of the use of these indicators to measure the performance of individual doctors. Conversely, some indicators (such as the cleanliness of a practice) were observed to have more variance at the practice level. For such indicators, organisations are in control, and these indicators are more suitable for the evaluation of performance at the level of the organisation. Our findings suggest that current practice level performance indicators, although they provide a potentially useful overview of average performance, may not provide meaningful information to commissioners, providers, or users for some key domains, such as communication skills. In particular, practices singled out as having lower performance through assessments of doctor-patient communication aggregated at a practice rather than an individual level are likely to contain a range of doctors. Patients attending such practices may see a general practitioner with excellent interpersonal skills or, alternatively, may see a doctor who is less proficient at communicating with patients. Conversely, the assessment of communication at the practice level may mask quite how poorly some general practitioners perform, as excellent doctors will pull up the average practice score. This has important implications for the ability to manage the performance of practices, as inadequate interpersonal skills might be missed. Finally, it is worth observing that patients may express choice through requests for continuity of care with a preferred doctor.

In compiling performance indicators to inform patients’ choices of providers, it would be preferable to report communication scores at the individual practitioner level or to report the range of individual practitioners’ scores within an organisation where that can be done reliably. Communication is a key driver of overall patients’ satisfaction,^18^ and ensuring patients’ ability to access accurate information on performance is important if they are expected to make informed choices among providers. If quality indicators are to be used to identify poor performance rather than to inform patients’ choice, an alternative to the potentially costly option of obtaining communication scores for all individual practitioners might be to use organisation level assessments such as those provided in the English GP Patient Survey to screen for lower performing practices. Where potential concerns about performance are identified by this mechanism, individual level assessments could then be targeted to those organisations alone. This approach, considering a low practice level score as a high sensitivity but low specificity test of whether a particular doctor in the practice may have a lower score in respect of communication, may be worthy of consideration, although determining reliable threshold scores for such a test would need data from a much larger sample than was available in this study. In addition, the cost effectiveness of such an approach remains to be determined, having never been explored in detail. Further research would be useful to explore the feasibility and practicality of alternative approaches to generating performance data on doctor-patient communication. More widely, many unanswered questions remain about the association between the publication of performance data and quality improvement, including the mechanisms underpinning any personal or organisational changes precipitated, and the perspectives of users, providers, and commissioners about the expected utility of alternative approaches.

### Conclusions

Current approaches to evaluating performance in communication frequently assess publicly reported indicators at an aggregate level, rather than enabling patients and other stakeholders to evaluate individual practitioners directly. Reporting communication related performance indicators at practice level may mask large variation between individual practitioners. Practice level surveys may offer potential to act as an initial screen for concerns about performance, with subsequent data gathering focusing on individual doctor level surveys in lower performing practices.

**What is already known on this topic**

Publication of performance data is increasingly the norm in healthcare systems, although evidence to support the mechanisms by which such publication may drive quality improvement is variable
Data collected to derive performance indicators may be collected at organisational (hospital, general practice) or individual practitioner level, with organisational level the norm for English general practice
For practitioner level performance indicators, such as the communication skills of individual doctors, variance within organisations is often greater than that between organisations

**What this study adds**

Practice level ratings of general practitioners’ consultation communication skills can mask considerable variation between the doctors within a practice, particularly in poorer performing practices
Patients registered with practices that have communication scores at the lower end of the spectrum may experience wide variation in communication skills between individual doctors
Higher scoring practices are very unlikely to include lower scoring doctors, but lower scoring practices require further investigation at the individual doctor level to distinguish higher and lower scoring general practitioners

## Notes

We thank the patients, practice managers, general practitioners, and other staff of the general practices who kindly agreed to participate in this study and without whom the study would not have been possible. Thanks also go to Emily Taylor, Jenny Newbould, Emma Whitton, Amy Gratton, Charlotte Paddison, and Dawn Swancutt for invaluable help with study set-up, practice recruitment, data collection, and data entry. We also thank the Improve Advisory Group for their input and support throughout this study. MJR is now senior psychometrician at the University of Plymouth Peninsula Schools of Medicine and Dentistry, Plymouth, UK.

Contributors: MJR wrote the statistical analysis plan, monitored data collection in the south west of England, analysed the data, and drafted and revised the paper. JLC designed the study, wrote the statistical analysis plan, oversaw the conduct of the study, and drafted and revised the paper. GAA contributed to the statistical analysis plan, carried out the sampling of practices, monitored data collection in the east of England/London, and revised the paper. AFD, IM, and MC carried out data collection in the south west, assisted with the data entry and commented on draft versions of the paper. MC and NLE assisted with ethics submissions. NLE carried out data collection in the east of England/London and commented on draft versions of the paper. MNE contributed to the statistical analysis plan and revised the paper. MOR designed the study, oversaw the conduct of the study, and revised the paper. JAB carried out and monitored data collection in the east of England/London, oversaw the conduct of the study, and drafted and revised the paper. JLC is the guarantor.

Ethical approval: Approval for the study was obtained from the South West 2 Research Ethics Committee on 28 January 2011 (ref: 09/H0202/65). Return of a completed questionnaire was taken to indicate patients’ consent to participate in the study.

Declaration of transparency: The lead author (study guarantor) affirms that this manuscript is an honest, accurate, and transparent account of the study being reported; that no important aspects of the study have been omitted; and that any discrepancies from the study as planned (and, if relevant, registered) have been explained.

Data sharing: No additional data available.

## Notes

Cite this as: *BMJ* 2014;349:g6034
