# Supplementary material for: Understanding high and low patient experience scores in primary care: analysis of patients’ survey data for general practices and individual doctors
Source: BMJ. 2014 Nov 11;349:g6034. doi: 10.1136/bmj.g6034 (PMC4230029; doi:10.1136/bmj.g6034)
Supplement: Supplementary file 2 — Appendix [file robm020243.ww2_default.pdf]

Appendix: Reliability of doctors' mean scores

Unit-level reliability is defined as the proportion of variance in reported unit sample means (e.g. practice means or physician means) attributable to true variation between units.[1] Due to the nesting of doctors within practices the reliability of the mean score for a randomly chosen doctor is calculated from the variance components of the three-level hierarchical model using the formula:

$$\text{Reliability} = [\text{var}(P) + \text{var}(D)] / [\text{var}(P) + \text{var}(D) + \text{var}(E)/N]$$

where var(P) = variance due to practices

var(D) = variance due to doctors

var(E) = variance due to patients and residual error

N = No. of patient scores per doctor

By inserting the values of the variance components and manipulating this formula the number of patient scores per doctor that are required to achieve a given level of reliability can be calculated. These calculations can be performed using variance components obtained from a model with no fixed effects where the resulting reliability is that pertaining to the raw mean scores. Alternatively, we can take into account the fact that some of the variation (at all levels) may occur due to different patient demographics and calculate a reliability of an adjusted mean score such as those shown in Figures 1 and 2. Both raw and adjusted reliabilities estimated from our data are shown in Table 4.

---

[1] Lyratzopoulos G, Elliott MN, Barbiere JM, Staetsky L, Paddison CA, Campbell JL, et al. How can Health Care Organizations be Reliably Compared?: Lessons From a National Survey of Patient Experience. *Med Care* 2011;**49**(8):724-33.
